# Supplementary material for: The causal relationship between the human gut microbiota and pyogenic arthritis: a Mendelian randomization study
Source: Front Cell Infect Microbiol. 2024 Nov 26;14:1452480. doi: 10.3389/fcimb.2024.1452480 (PMC11629706; doi:10.3389/fcimb.2024.1452480)
Supplement: Supplementary file 1 [file DataSheet1.zip › mendelian test/ebi-a-GCST90027793.csv_forest.pdf]

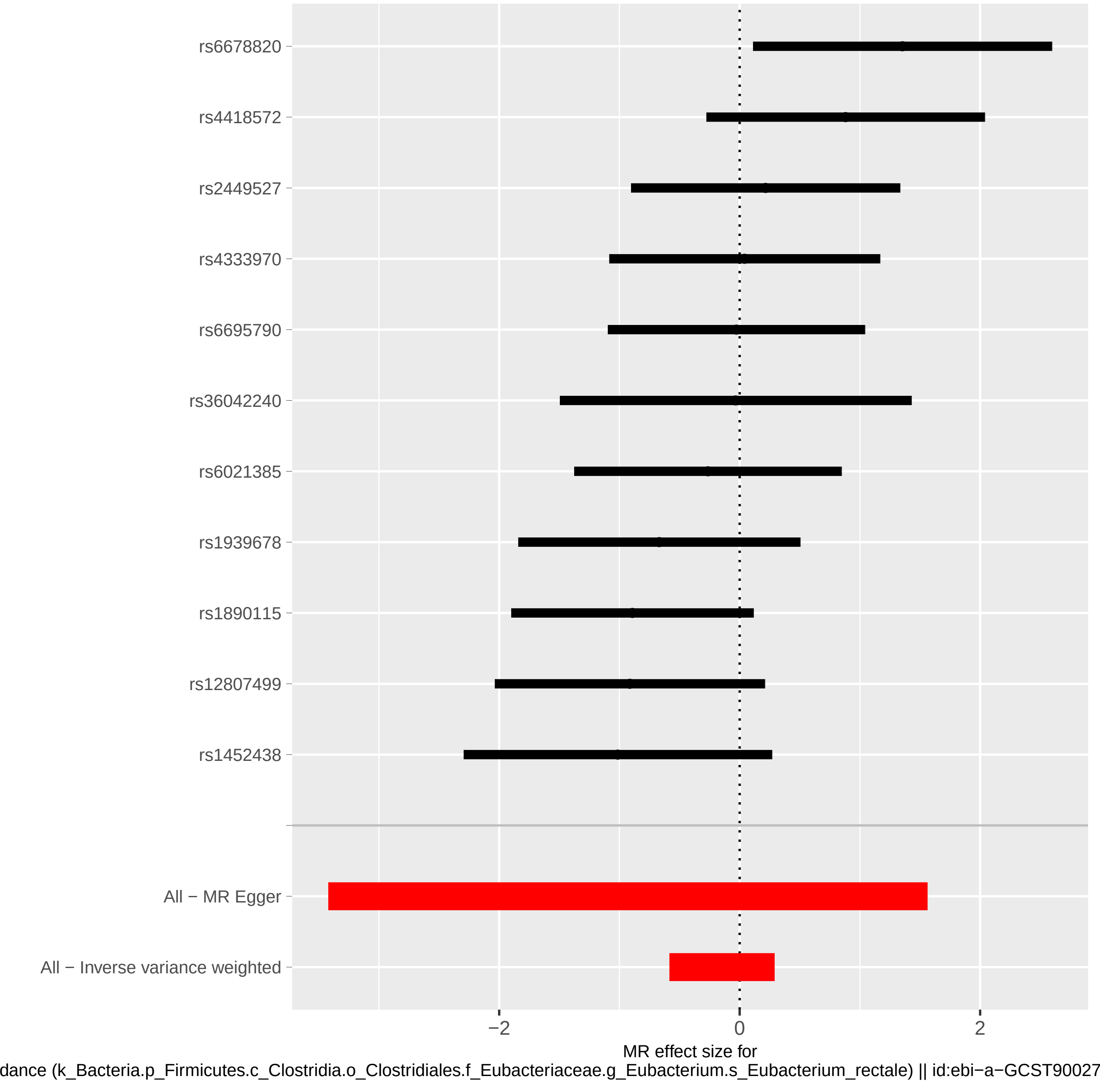
rs6678820
rs4418572
rs2449527
rs4333970
rs6695790
rs36042240
rs6021385
rs1939678
rs1890115
rs12807499
rs1452438
All − MR Egger
All − Inverse variance weighted
−2
0
2
MR effect size for
dance (k_Bacteria.p_Firmicutes.c_Clostridia.o_Clostridiales.f_Eubacteriaceae.g_Eubacterium.s_Eubacterium_rectale) || id:ebi−a−GCST90027
